# Supplementary material for: Specific Patterns of Blood ILCs in Metastatic Melanoma Patients and Their Modulations in Response to Immunotherapy
Source: Cancers (Basel). 2021 Mar 22;13(6):1446. doi: 10.3390/cancers13061446 (PMC8004602; doi:10.3390/cancers13061446)
Supplement: Supplementary file 1 [file cancers-13-01446-s001.pdf]

Table S1. List of mAbs used in the studies.

Marker	Clone	Source	Identifier
CD3	OKT3	Biolegend	317306
CD4	RPA-T4	BD Bioscience	555346
CD5	UCHT2	Biolegend	300606
CD33	HIM3-4	Biolegend	303304
CD14	M5E2	BD Bioscience	555397
CD19	HIB19	Biolegend	302206
TCRAB	IP26	Biolegend	306706
TCRGD	B1	Biolegend	331208
CD235a	HI264	Biolegend	349104
CD7	M-T701	BD Bioscience	562541
CD127	MB15-18C9	Miltenyi Biotec	130-113-410
CD56	BD159	BD Bioscience	557919
CD16	3G8	BD Bioscience	560195
CD117	104D2	BD Bioscience	562687
CRTH2	BM16	Biolegend	350104
NKp46	9E2/NKp46	BD Bioscience	562101
NKp30	P30-15	BD Bioscience	563385
NKG2D	1D11	BD Bioscience	563408
DNAM-1	DX11	Miltenyi Biotec	130-099-966
NKG2A	131411	BD Bioscience	747917
TIGIT	741182	BD Bioscience	747840
CTLA4	BNI3	BD Bioscience	555854
CD69	FN50	BD Bioscience	612817
CD62L	145/15	Miltenyi Biotec	130-113-619
CD96	NK92.39	Biolegend	338411
CD161	HP-3G10	BD Bioscience	748280
